# 3,3′-Diindolylmethane Enhances Fluorouracil Sensitivity via Inhibition of Pyrimidine Metabolism in Colorectal Cancer

**DOI:** 10.3390/metabo12050410

**Published:** 2022-04-30

**Authors:** Jieping Zhang, Shaomin Zou, Yijing Zhang, Ziqing Yang, Wencong Wang, Manqi Meng, Junyan Feng, Peng Zhang, Lishi Xiao, Mong-Hong Lee, Lekun Fang

**Affiliations:** Guangdong Provincial Key Laboratory of Colorectal and Pelvic Floor Disease, Guangdong Research Institute of Gastroenterology, The Sixth Affiliated Hospital of Sun Yat-sen University, Guangzhou 510000, China; zhangjp59@mail2.sysu.edu.cn (J.Z.); zoushm5@mail2.sysu.edu.cn (S.Z.); zhangyj78@mail2.sysu.edu.cn (Y.Z.); yangzq5@mail2.sysu.edu.cn (Z.Y.); wangwc5@mail.sysu.edu.cn (W.W.); mengmq3@mail.sysu.edu.cn (M.M.); fengjyan@mail3.sysu.edu.cn (J.F.); zhangp235@mail.sysu.edu.cn (P.Z.); xiaolsh6@mail.sysu.edu.cn (L.X.)

**Keywords:** colorectal cancer, 3,3′-Diindolylmethane, chemosensitivity, pyrimidine metabolism

## Abstract

Chemoresistance limits treatment outcomes in colorectal cancer (CRC) patients. A dimeric metabolite of indole-3-carbinol, 3,3′-diindolylmethane (DIM) is abundant in cruciferous vegetables and has shown anticancer efficacy. The role of DIM in regulating chemosensitivity in CRC remains unknown. In this study, we demonstrated that DIM treatment inhibits the malignant progression of CRC. RNA sequencing indicated that pyrimidine synthesis genes are attenuated by DIM treatment. Stable 1^3^C-labeled glucose tracing revealed that DIM inhibits de novo pyrimidine biosynthesis in CRC. DIM increases 5-FU cytotoxicity in CRC via regulation of the expression of pyrimidine metabolism-related genes. DIM synergizes with 5-FU to enhance its inhibitory effects on CRC both in vivo and in vitro. Our results suggest that DIM improves the therapeutic outcomes of FU-based chemotherapy in CRCs by inhibiting pyrimidine metabolism, identifying a new strategy for clinical therapy.

## 1. Introduction

Colorectal cancer (CRC) is a major digestive tract cancer that is prevalent worldwide [1,2]. Surgery, radiotherapy, and chemotherapy are the main therapeutic options for colorectal cancer [3,4]. Chemotherapeutic drugs induce cell cycle arrest and DNA repair deficiency to disrupt cell progression. Patients with stage II, stage III, and stage IV CRC exhibit increased benefit after receiving chemotherapy [5]. The first-line chemotherapeutic agent for CRC, 5-fluorouracil (5-FU), targets nucleotide metabolism and inhibits cancer cell growth either by binding to thymidylate synthase or by incorporating into RNA and DNA [6]. However, acquired resistance to 5-FU limits the use of this drug and has resulted in the development of novel treatments [7,8]. Presumably, targeting nucleotide metabolism is an effective strategy to improve the prognosis of patients. Therefore, combination chemotherapeutic regimens are a promising method to improve treatment.

Emerging evidence suggests that diet is linked to CRC prevention, carcinogenesis, and survival, but the underlying mechanisms still need more investigation [9，^10^，^11^]. Environmental factors, such as diet, are considered to account for the incidence of CRC in some patients [2]. Numerous bioactive substances derived from the diet exert various biological effects in different diseases. Recently, remarkable achievements have been made in combination therapy with natural compounds and chemotherapeutic drugs in CRC [12]. Curcumin and FOLFOX exert synergistic effects in CRC cell lines [13], apigenin potentiates 5-FU-induced cytotoxicity [14], and epigallocatechin-3-gallate enhances 5-FU efficacy by maintaining an increased intracellular concentration of 5-FU [15]. DIM is a dimeric metabolite of the natural product indole-3-carbinol. DIM is widely found in cruciferous vegetables (broccoli, cabbage, cauliflower, etc.). Accumulating evidence indicates that DIM exerts exceptional biological effects. An ins vitro binding assay suggested that DIM can directly bind to COX 1/2 and ERK 1/2, which subsequently inhibits tumor development in CRC [16]. DIM decreases the expression of class I HDACs to induce cell cycle arrest and apoptosis [17]. DIM can maintain intestinal barrier homeostasis [18]. However, it is still not clear whether DIM correlates with the efficacy of chemotherapy.

Pyrimidines are involved in various biological processes and are constantly synthesized through endogenous mechanisms in cancer cells. Six steps are required for de novo pyrimidine metabolism, and the process is regulated at multiple levels [19]. Normally, the cellular pyrimidine pool is maintained through the de novo synthesis and salvage pathways, and proliferating cells such as cancer cells preferentially use the de novo synthesis pathway [20]. Pyrimidine nucleotides are indispensable substrates for DNA and RNA synthesis, and growth signaling increases de novo pyrimidine synthesis to support cancer cell growth [21]. Cytidine triphosphate (CTP) is a precursor for cellular phospholipid biosynthesis and is involved in cellular component organization and signal transduction [22]. Uridine triphosphate (UTP) participates in the production of glycosaminoglycans and is employed in glycosylation modification of proteins [23,24]. Hence, depleting nucleotide pools has long been considered a feasible option for CRC treatment. The stable isotope tracing method allows the monitoring of isotopically enriched precursors contributing to biological processes in cancer cells [25]. Coupled with mass spectrometry, stable isotope tracing has facilitated investigation of the potential mechanism of DIM and the targeting of cancer cell metabolism.

The objective of this study was to investigate the synergistic effect of DIM and 5-FU and to elucidate the potential molecular mechanisms. We analyzed the effects of DIM alone on proliferation, cell cycle, apoptosis, and ROS production of CRC cells. Furthermore, we confirmed its antitumor effect in vivo. Based on RNA-seq data and ^13^C-glucose stable isotope tracing, we confirmed the alteration of pyrimidine metabolism under DIM treatment. The effects of the combination of DIM and 5-FU on CRC cell viability, clonality, and apoptosis were analyzed in vitro. Finally, the combination efficacy of DIM and 5-FU was evaluated in vivo. Our efficacy studies on the combination of DIM and 5-FU reveal a novel therapeutic strategy for colorectal cancer.

## 2. Results

### 2.1. DIM inhibits CRC Progression In Vitro and In Vivo

**Figure 1:**
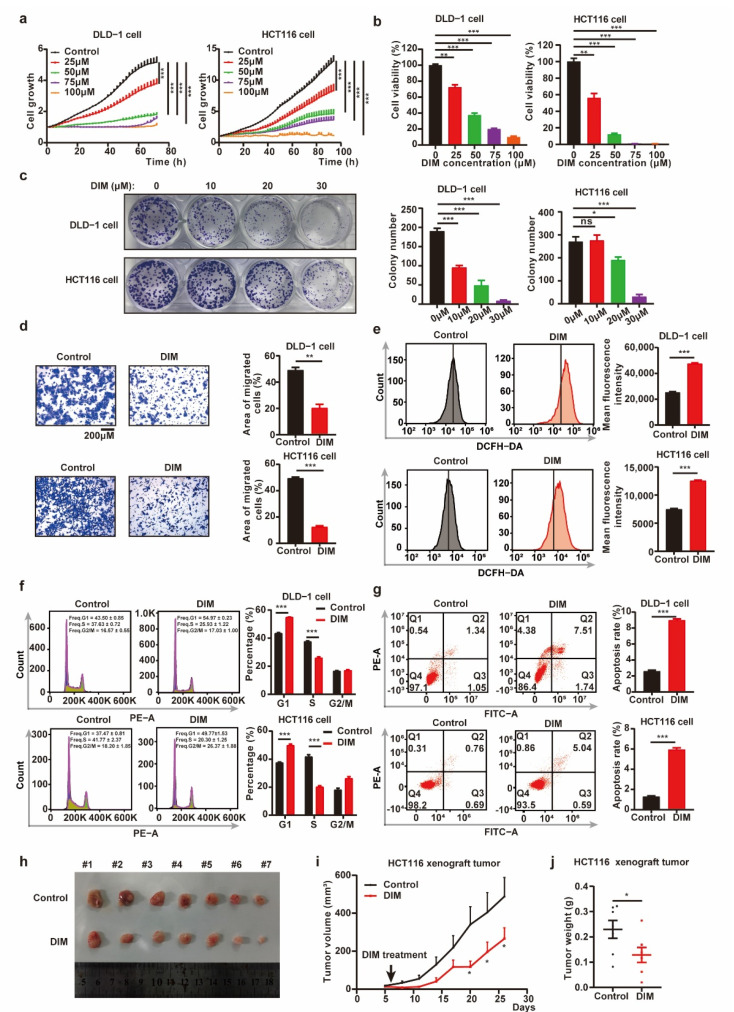
DIM inhibits CRC progression in vitro and in vivo. (**a**) The growth curve for CRC cells treated with various concentrations (0, 25, 50, 75, 100 μM) of DIM were obtained by IncuCyte machine. (**b**) Cell viability of CRC cells was detected using CCK-8 assay. CRC cells were treated DIM (0, 25, 50, 75, 100 μM) for 48 h. (**c**) Colony formation experiments of DLD-1 and HCT116 cells after treatment with DIM (0, 10, 20, 30 μM) for 9 days. (**d**) DLD-1 and HCT116 cells were treated with or without DIM (40 μM). Cell migrations were determined using transwell assays. Scale bar: 200 μM. (**e**) Flow cytometry analyzed the ROS production influenced by DIM in CRC cells. DLD-1 and HCT116 cells were treated with or without DIM (40 μM) for 48 h. (**f**) Cell cycle analysis of DLD-1 and HCT116 cells treated with DIM (40 μM) for 48 h. (**g**) Effects of DIM treatment on cell apoptosis. DLD-1 and HCT116 cells were treated with DIM (40 μM) for 48 h. Representative flow cytometric histogram images show the effects of DIM treatment. (**h**) Representative images of tumors in control (*n* = 7) and DIM (*n* = 7) HCT116 xenograft groups. (**i**) Tumor growth curves comparing control (*n* = 7) and DIM (*n* = 7) HCT116 xenograft every three days were plotted. (**j**) Measured weights of tumors in control (*n* = 7) and DIM (*n* = 7) HCT116 xenograft groups. Data are presented as mean ± SEM. ns, no significance; *, *p* < 0.05; **, *p* < 0.01; ***, *p* < 0.001; unpaired two-tailed Student test, one-way ANOVA test, and two-way ANOVA test.

Previous studies have shown that DIM exerts antitumor effects in different cancer types, including CRC [17,26]. To further investigate the potential antitumor mechanisms of DIM, we treated CRC cells with different concentrations of DIM. The cell growth curves obtained by IncuCyte analysis and cell viability from CCK-8 assay indicated that DIM reduced the viability and proliferation of CRC cells compared with control cells in a dose-dependent manner (Figure 1a,b). Consistent with the growth curves, DIM treatment resulted in an inhibitory effect on colony formation, while the sensitivity of CRC cell lines to DIM varied (Figure 1c). In addition, the role of DIM in cell migration was analyzed using transwell assays. The results suggested that CRC cell migration was inhibited by DIM compared to the control group (Figure 1d). In this study, flow cytometry was performed to calculate the ROS level. The results showed that DIM treatment led to an increase in the production of ROS (Figure 1e). Moreover, propidium iodide (PI) staining was conducted to evaluate the impact of DIM on the cell cycle phase distribution. The results showed that DIM induced G1 arrest in DLD-1 cells and HCT116 cells (Figure 1f). In addition, DLD-1 cells and HCT116 cells were treated with DIM for 48 h, and Annexin V-FITC/PI staining was performed to determine the effect of DIM on apoptosis. Flow cytometric analysis revealed that DIM induced apoptosis in both DLD-1 and HCT116 cells (Figure 1g).

To further confirm the potential antitumor efficacy of DIM, the growth of HCT116 cells was analyzed in vivo in a xenograft model established by subcutaneous injection of cells. As shown in Figure 1h–j, treatment with DIM decreased the tumor volumes and tumor weights compared with those in the control group. Collectively, these results imply that DIM exerts an antitumor effect in CRC.

### 2.2. DIM Treatment Attenuates Pyrimidine Metabolism in CRC Cells

**Figure 2:**
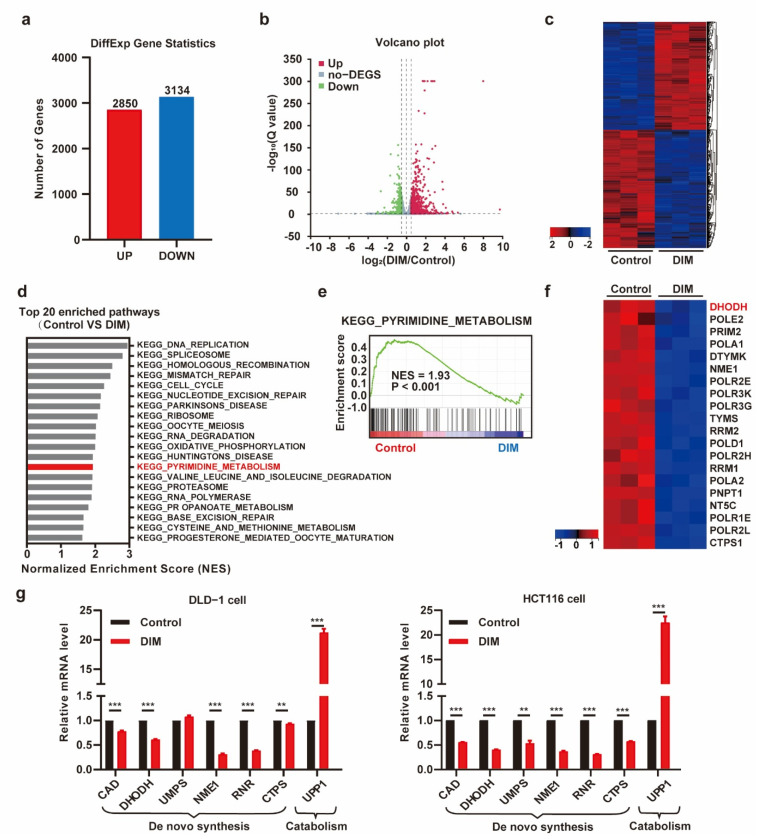
DIM treatment attenuates the pyrimidine metabolism in CRC. (**a**) Statistics for both up- and down-regulated number of the differently expressed genes (DEGs). (**b**) The volcano plot of differential expression genes in DMSO- and DIM-treated DLD-1 cells (|logFC| > 0.5 and *p*-value < 0.05). (**c**) Heatmap plot of gene expression levels based on RNA-seq data by Sangerbox platform. Data colored in the red–black–blue scheme indicate a relatively higher, average, and lower concentration, respectively. (**d**) Representative top 20 pathways enriched in control group compared to DIM group based on GSEA analysis results. (**e**) Enrichment plots for KEGG_PYRIMIDINE_METABOISM are shown. (**f**) Heatmap plot of top 20 genes ranked in KEGG_PYRIMIDINE_METABOISM. (**g**) DIM dysregulated the transcription of genes in pyrimidine metabolism (CAD, DHODH, UMPS, NME1, RNR, CTPS, UPP1) by RT-PCR. DLD-1 cells and HCT116 cells were treated with 40 μM DIM for 48 h. Data are presented as mean ± SEM. **, *p* < 0.01; ***, *p* < 0.001; unpaired two-tailed Student *t*-test.

The potential molecular mechanisms of DIM were studied by mRNA sequencing and analysis of differentially expressed genes (DEGs). We treated DLD-1 cells with medium containing DIM and profiled their gene expression. Analysis of gene expression after DIM intervention revealed that DIM changed the gene expression profile; in total, 2850 genes were upregulated, and 3134 genes were downregulated (Figure 2a). The heatmap and volcano plot helped to visualize the DEGs and identified different signatures (Figure 2b,c). We further performed gene set enrichment analysis (GSEA), a computational approach to determine the biological difference between groups. The top 20 pathways significantly enriched with numerous DEGs in each biological process are listed in Figure 2d. The pyrimidine metabolism pathway was enriched in the control group (Figure 2e). The top 20 genes in the “KEGG_PYRIMIDINE_METABOLISM” pathway are also listed (Figure 2f). Furthermore, the expression levels of some genes related to pyrimidine metabolism were confirmed by real-time PCR. DIM inhibited the expression of genes associated with pyrimidine synthesis, while the expression of the pyrimidine-catabolism-associated gene UPP1 was increased (Figure 2g). Taken together, these results indicate that DIM treatment induces disruption of pyrimidine metabolism.

### 2.3. Metabolic Profiling Reveals de Novo Pyrimidine Biosynthesis Alteration in CRC under DIM Treatment

**Figure 3:**
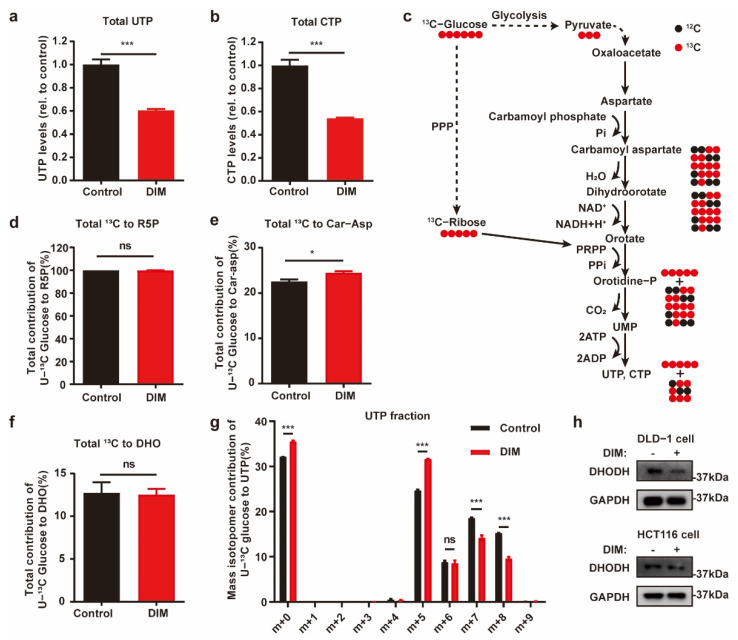
Metabolic profiling reveals de novo pyrimidine biosynthesis alteration in CRC under DIM treatment. (**a**) Total content of UTP in control and 40 μM DIM-treated HCT116 cells. (**b**) Intracellular CTP levels in control and 40 μM DIM-treated HCT116 cells. (**c**) Schematic overview of ^13^C-glucose-traced pyrimidine biosynthesis. Black circle: ^12^C; red circle: ^13^C. (**d**) Total labeled carbons in ribose-5-phosphate (R5P) of control and DIM-treated HCT116 cells following a ^13^C-glucose treatment. (**e**) Total labeled carbons in Car-Asp of control and DIM-treated HCT116 cells following a ^13^C-glucose treatment. (**f**) Total labeled carbons in DHO of control and DIM-treated HCT116 cells following a ^13^C-glucose treatment. (**g**) Fraction enrichment of ^13^C-glucose derived isotopologues in UTP in control and 40 μM DIM-treated HCT116 cells. (**h**) Western blot analysis after DIM treatment using anti-DHODH. GAPDH was used as the internal control in DLD-1 and HCT116 cells. Data are presented as mean ± SEM. ns, no significance; *, *p* < 0.05; ***, *p* < 0.001; unpaired two-tailed Student *t*-test.

To confirm whether pyrimidine synthesis is blocked by DIM treatment in CRC, we analyzed the total contents of UTP and CTP by using mass spectrometry. Following DIM treatment, the total contents of UTP and CTP in HCT116 cells were decreased (Figure 3a,b). Furthermore, we performed ^13^C-glucose tracing to compare the metabolite profiles of control and DIM-treated HCT116 cells. A schematic overview of de novo pyrimidine synthesis and the use of ^13^C-labeled glucose to monitor pyrimidine synthesis is shown in Figure 3c. Generally, glucose carbon is incorporated into aspartate, which is condensed with carbamoyl phosphate (Car-Asp) to form dihydroorotate (DHO), which is further oxidized to orotate via dihydroorotate dehydrogenase (DHODH). Glucose carbon is also incorporated into pyrimidine nucleotides via several reactions catalyzed by phosphoribosyl pyrophosphate. Phosphoribosyl pyrophosphate is added to yield orotidine monophosphate, from which UMP, UTP, and CTP are produced.

Across all the tested conditions, the utilization of PPP branch-derived ribose-5-phosphate for pyrimidine biosynthesis was not obviously changed among the metabolome pools, suggesting that carbon flux from the PPP is not the dominant pathway for the cytotoxic response to DIM in CRC cells (Figure 3d). When comparing the metabolic differences of aspartate-derived pyrimidine nucleotides, we found slightly elevated enrichment of Car-Asp, while there was no difference in the DHO content (Figure 3e,f). We also found that the distribution of UTP (m + 6 to m + 9) was decreased in DIM-treated cells (Figure 3g). Accordingly, the metabolic characteristics of UTP confirmed that the decreased levels of pyrimidine nucleotides in DIM-treated CRC cells were due to inhibited synthesis of aspartate-derived pyrimidine nucleotides. Consistent with our findings, the results of real-time PCR, GSEA, and Western blotting revealed that inhibition of DHODH was consistent among the cell lines (Figure 2f,g and Figure 3h).

### 2.4. DIM Promotes Chemosensitivity of 5-FU and Potentiates 5-FU-Induced Suppressive Effects on CRC Cells

**Figure 4:**
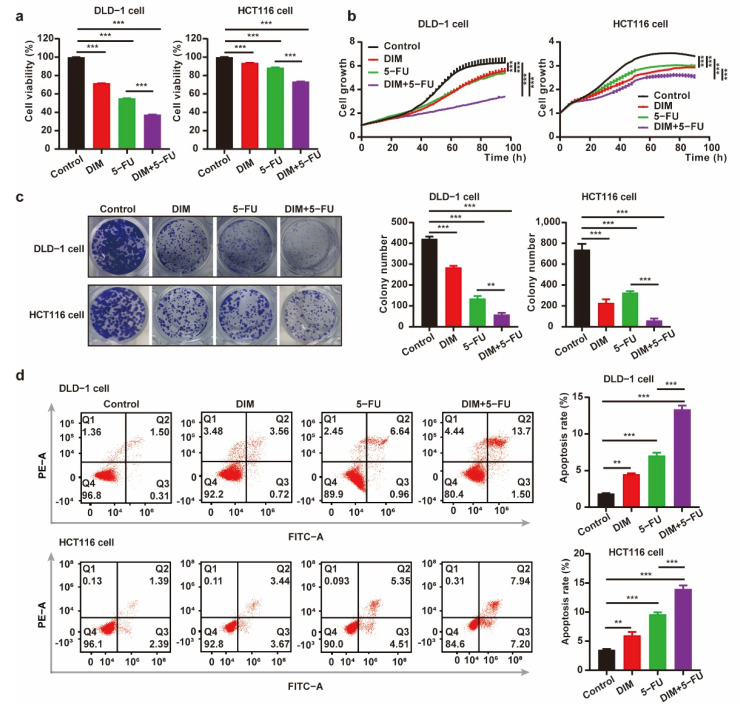
DIM increases the chemosensitivity to 5-FU and potentiates 5-FU-induced suppressive effect on CRC cells. (**a**) Cell viability of CRC cells was detected using CCK-8 assay. DLD-1 cells (DIM, 30 μM; 5-FU, 10 μM) and HCT116 cells (DIM, 20 μM; 5-FU, 2.5 μM) were treated with DIM for 48 h. (**b**) Relative cell growth in CRC cells treated with DIM, 5-FU, or the combination of DIM and 5-FU. DLD-1 cells (DIM, 30 μM; 5-FU, 10 μM) and HCT116 cells (DIM, 20 μM; 5-FU, 2.5 μM) were monitored throughout the whole process by the IncuCyte machine. (**c**) A colony formation assay treated with DIM and 5-FU alone or in combination was carried out using CRC cells. DLD-1 cells (DIM, 30 μM; 5-FU, 10 μM) and HCT116 cells (DIM, 20 μM; 5-FU, 2.5 μM) were plated in 12-well plates for 9 days. (**d**) Flow cytometry analysis of apoptosis in CRC cells pretreated with DIM and 5-FU (DLD-1 cell: 30 μM DIM and 10 μM 5-FU; HCT116 cell: 20 μM DIM and 2.5 μM 5-FU) for 48 h. The percentage value of Q2 + Q3 indicates the proportion of apoptotic cells. Data are presented as mean ± SEM. **, *p* < 0.01; ***, *p* < 0.001; two-way ANOVA test.

Our objective was to study the synergistic effect of DIM and 5-FU on CRC cytotoxicity. It has been reported that 5-FU targets pyrimidine metabolism. Therefore, we sought to determine whether there is synergism between DIM and 5-FU. We performed combination treatment with 5-FU and DIM in different CRC cell lines. As shown in Figure 4a,b, in comparison to 5-FU or DIM alone, the combination of 5-FU and DIM showed potent inhibitory effects on cancer cell viability and proliferation. Furthermore, CRC cells were treated with the indicated concentrations of 5-FU and DIM alone or in combination for evaluation of colony formation. Consistent with the results of the cancer cell growth assay, the combination of 5-FU and DIM reduced the capacity for colony formation (Figure 4c). Similar results were also observed in the apoptosis assay. Combination treatment with DIM and 5-FU notably increased the number of apoptotic cells, and all results were further quantified (Figure 4d). Collectively, these results indicate that DIM increases the therapeutic effect of 5-FU in vitro.

### 2.5. DIM Enhances the Effects of 5-FU-Based Chemotherapy In Vivo

**Figure 5:**
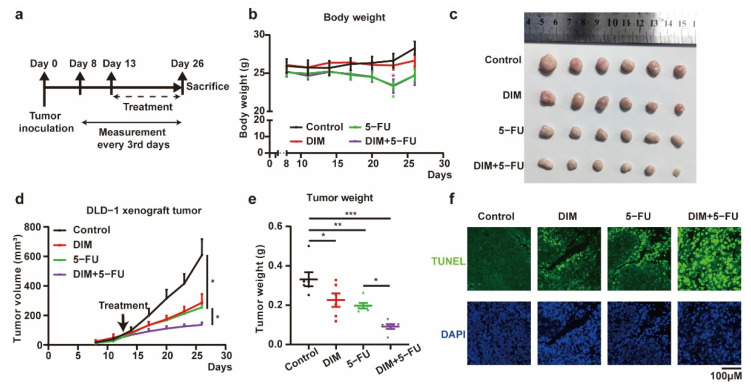
DIM enhances 5-FU-based chemotherapy effects in vivo. (**a**) Schematic overview of in vivo experiment. Tumor volumes and body weights were measured every three days from the eighth day after tumor inoculation. Intraperitoneal injection 6 times with DIM (40 mg/kg, 10%DMSO) and 3 times with 5-FU (40 mg/kg, PBS) from the thirteenth day after tumor inoculation. (**b**) Representative images of tumor in control (*n* = 6), DIM (*n* = 6), 5-FU (*n* = 6), and DIM + 5-FU (*n* = 6) groups were plotted. (**c**) Measured body weights of mice in control, DIM, 5-FU, and DIM + 5-FU groups. (**d**) Tumor growth curves comparing control, DIM, 5-FU, and DIM + 5-FU DLD-1 xenograft every three days were plotted. (**e**) Corresponding tumor weights in control, DIM, 5-FU, and DIM + 5-FU groups were plotted. (**f**) Xenograft tumor sections were fixed to analyze the apoptotic cells (green) by TUNEL staining (green). Representative images are shown. Scale bar: 100 μm. Data are presented as mean ± SEM. *, *p* < 0.05; **, *p* < 0.01; ***, *p* < 0.001; Two-way ANOVA test.

To confirm the in vitro findings described above, the synergistic effects of combined treatment with DIM and 5-FU were examined in a mouse xenograft model (Figure 5a). Loss of body weight was observed in the groups that received 5-FU, and DIM had no protective effects on body weight (Figure 5b). The volume and weight of tumors were decreased in the groups that received DIM, 5-FU, or both DIM and 5-FU. Additionally, the therapeutic efficacy of 5-FU was improved by DIM (Figure 5c–e). Subsequently, we determined the apoptosis rate of DLD-1 cells in the tumor mass after different treatments. Single-agent treatment with DIM or 5-FU induced apoptosis, and combination treatment with DIM and 5-FU potentiated the effect of each single agent on apoptosis (Figure 5f). Consistent with the in vitro results, DIM treatment increased the cytotoxic response to 5-FU in CRC xenograft tumors.

## 3. Discussion

Treatment with adjuvant chemotherapy is a therapeutic strategy currently used for patients with CRC. CRC patients who received adjuvant chemotherapy showed prolonged median overall survival times and disease-free survival times [27]. Note that 5-FU is a first-line chemotherapeutic drug for CRC patients, but continuous treatment with 5-FU always results in the development of chemoresistance, which limits therapeutic efficacy [28]. Natural compounds obtained through the diet are currently known to exert antitumor effects, and interestingly, DIM derived from cruciferous vegetables is a potential antitumor compound [29]. Accumulating evidence indicates that DIM can regulate the malignant progression of various tumors both in vivo and in vitro by participating in different signaling mechanisms [30]. However, whether DIM can restore 5-FU sensitivity in CRC remains unclear, and further analysis is needed. Hence, investigating the effects of DIM in combination with 5-FU has clinical value.

Pyrimidine-metabolism-associated metabolic enzymes, cancer cell stemness, MDR transporters, and the tumor microenvironment are factors that affect sensitivity to 5-FU. Combination therapy with natural compounds is a strategy to enhance sensitivity to 5-FU in CRC [31]. In this study, we revealed a novel function of DIM in regulating pyrimidine metabolism and 5-FU cytotoxicity in CRC cells. The previous studies indicate that DIM can have either favorable or unfavorable effects in different types of disease. For example, DIM has been reported to act as either an antioxidant or a pro-oxidant. DIM can protect against adriamycin-induced cardiac fibrosis via activation of an antioxidant pathway involving BRCA1 expression [32]. DIM alleviates radiation-induced intestinal injury by increasing the activities of antioxidant enzymes [18]. A previous study indicated the pro-oxidant effect of DIM under hypoxic conditions in breast cancer [33]. Our study showed a significant increase in ROS production after DIM treatment; thus, we speculated that DIM created a hypoxic environment and then induced ROS accumulation. The chemopreventive effects of DIM have been partially elucidated, but further study is needed. These effects could be mediated through modulation of cancer cell apoptosis, estrogen metabolism, proliferation inhibition, and cell cycle arrest [17,34,35]. These results are consistent with previously published cytotoxicity assays in CRC. In this study, DIM treatment reduced the capability for cell growth and induced G1 arrest and apoptosis. The findings in the in vivo xenograft model also confirmed the inhibitory effects of DIM. Next, RNA sequencing was performed to determine gene expression levels after DIM treatment. Potential biological pathways related to the cytotoxic response to DIM were identified by GSEA. The results indicated that these genes were mainly enriched in the pyrimidine metabolism pathway in the nontreated groups. Targeting pyrimidine metabolism in cancer cells is often considered a feasible therapeutic strategy [36]. We further confirmed the dysregulation of the de novo pyrimidine synthesis pathway and catabolic pathway using real-time PCR. The results shown that DIM inhibited pyrimidine synthesis and accelerated the catabolism of pyrimidine nucleotides. Stable isotope tracing also implied the inhibition of de novo pyrimidine biosynthesis and the reduction in pyrimidine nucleotide pools. Combining all these data from real-time PCR and stable isotope tracing, we hypothesized that DHODH is the hub gene that mediates DIM-induced inhibition of pyrimidine biosynthesis. A previous study revealed that pyrimidine metabolism is a factor mediating the response to 5-FU [37]. Therefore, we speculated that DIM has synergism with 5-FU. To confirm our hypothesis, DIM and 5-FU alone or in combination were used to treat CRC cells. We found that DIM combined with 5-FU reduced cell viability and the proliferation rate but elevated 5-FU-induced apoptosis. Additionally, DIM may enhance 5-FU efficacy by further reinforcing the inhibition of pyrimidine synthesis. These results were further supported by xenograft experiments, where the lower tumor weights and reduced growth indicated a potentiated inhibitory effect in the combination group compared to the other groups. Further analysis is therefore required to investigate the potential molecular mechanism underlying the effects of DIM on the suppressive effects of 5-FU in CRC cells.

DHODH is a mitochondrial enzyme involved in pyrimidine synthesis; it is responsible for the conversion of DHO to orotate and is related to electron transport. Overexpression of DHODH is observed in various cancers, and DHODH acts as an oncogene in some cancer types [38,39]. The commonly studied role of DHODH is as a metabolic enzyme for pyrimidine synthesis, and inhibition of DHODH results in disruption of the cellular pyrimidine profile. Previous studies have shown that inhibition of DHODH by compounds is a potential molecular mechanism by which the cytotoxic response to 5-FU is enhanced [40,41]. In our study, we demonstrated that DIM inhibits DHODH-dependent pyrimidine metabolism and that the combination of DIM and 5-FU exhibits increased therapeutic efficacy. COX1/2, similar to DHODH, are enzymes involved in mitochondrial electron transport. Study have reported that DIM can directly bind to COX 1/2 and ERK 1/2 proteins, which subsequently inhibits tumor development in CRC [16]. We may explore whether COX 1/2 and ERK 1/2 participate in DIM regulation on pyrimidine metabolism in the future.

## 4. Materials and Methods

### 4.1. Cell Culture

The human DLD-1 and HCT116 colon cancer cell lines were purchased from the American Type Culture Collection (ATCC) and propagated and passaged as adherent cell cultures according to instructions provided by ATCC. All cells were maintained in a humidified incubator with 5% CO_2_ at 37 °C. HCT116 and DLD-1 cells were maintained in RPMI/1640 (Corning Life Sciences, Corning, NY, USA), supplemented with 10% (*v*/*v*) fetal bovine serum (FBS, GIBCO, Grand Island, NY, USA) and 1% penicillin–streptomycin. The medium was changed every other day, and cells were passaged using 0.25% trypsin/EDTA (Corning Life Sciences, Corning, NY, USA).

### 4.2. Reagents and Antibodies

The 3,3’-Diindolylmethane (D129118-100 g) and 5-Fluorouracil (F100149-25 g) were purchased from Aladdin (Shanghai, China). Corn oil (S50856) was obtained from Shanghai Yuanye Bio-Technology Co., Ltd. (Shanghai, China). DMSO (Q6949) was from MP Biomedicals (Santa Ana, CA, USA). Mouse anti-GAPDH (60004-1-Ig) was from Proteintech Group (Wuhan, China). Rabbit anti-DHODH (14877-1-AP) was obtained from Proteintech Group (Wuhan, China).

### 4.3. Western Blotting

After washing with PBS three times, cells were harvested. Collected cells were treated with RIPA lysis buffer (50 mM Tris-HCl pH 7.5, 150 mM NaCl, 1% NP-40, 0.1% SDS, 5 mM EDTA, 1 mM NaF) with phosphatase and protease inhibitor cocktail (B15002 and B14002, BIMAKE, Houston, TX, USA), and sonicated to release the contents. The protein concentrations were determined using Bestbio BCA protein assay kit (BB-3401-3, Shanghai, China). Total protein (10 µg) was separated by 10% SDS-PAGE and transferred to polyvinylidene fluoride membranes (Millipore, Boston, MA, USA). After incubation in 5% non-fat milk (A600669-0250, Sangon Biotech, Shanghai, China) solution for one hour, the membranes were incubated in the primary antibodies GAPDH (1:5000) and DHODH (1:8000) at 4 °C overnight. The proteins were visualized by using the ECL Reagent (170-5061, Bio-Rad, Hercules, CA, USA) on ChemiDoc Touch Imaging system (Bio-Rad, Hercules, CA, USA).

### 4.4. CCK-8 Assay

Cell viability was determined by Cell Counting Kit-8 (K1080, Apexbio Technology LLC, Houston, TX, USA). CRC were seeded into 96-well plates or 24-well plates for 24 h. The cells were treated with indicated doses of DIM, 5-FU, and the combination of DIM and 5-FU for 48 h. Then, 10% (*v*/*v*) CCK-8 solution was prepared and added to every well, and cells were plated into a cell incubator for 3 h [42]. Optical density was measured at 450 nm using Epoch™ Multi-Volume Spectrophotometer and Take3™ (BioTek, Winooski, VT, USA). Cell viability was normalized as a percentage of the negative controls treated with DMSO.

### 4.5. Growth Curve

Cell viability was monitored by the IncuCyte live cell analysis system (Essen BioScience, Ann Arbor, MI, USA). To assess the cell proliferation, CRC cells were equally seeded into fresh 12-well plates at a density of 1 × 10^5^ cells. Cells were treated with indicated concentrations of DIM, 5-FU, or DIM and 5-FU after 6 h of adherent culture. After placing the cell in a cell incubator, the IncuCyte live cell analysis system will automatically obtain and analyze the photographs at 9 or 16 random fields.

### 4.6. Colony Formation

The DLD-1 and HCT116 cells were seeded into 12-well plates (BD Falcon, Franklin Lakes, NJ, USA) at a density of 500 cells per well. To determine the antitumor effect of DIM, cells were treated with DIM (0, 10, 20, 30 μM) after 2 days of adherent culture, with 9 days of maintenance in RPMI medium 1640 (HCT116 and DLD-1 cell) supplemented with 10% FBS and 1% penicillin and streptomycin. To determine the synergistic effect of DIM and 5-FU, after 2 days of adherent culture, cells were treated with 5-FU (HCT116, 2.5 μM; DLD-1, 10 μM), DIM (HCT116, 20 μM; DLD-1, 30 μM), or both 5-FU and DIM, with 9 days of maintenance in RPMI medium 1640 supplemented with 10% FBS and 1% penicillin and streptomycin. After washing three times with PBS, the colonies were fixed with 4% polyoxymethylene (15 min) and stained with 0.05% crystal violet (10 min) at room temperature. Number of colonies was calculated by Imagej (NationalInstitutes of Health, Bethesda, MD, USA).

### 4.7. Cell Migration Assay

Cell transwell experiments were carried out using 8.0 µm PET track-etched membrane transwell (BD Falcon, Franklin Lakes, NJ, USA), with each transwell inserted into the 24-well plates (NEST, Shanghai, China) to form an upper and a lower chamber. DLD-1 and HCT116 cells were pretreated with DIM (0, 40 μM) for 48 h. To elucidate the effect of DIM on migration ability, serum-free medium (200 µL) containing 2 × 10^5^ DLD-1 and HCT116 cells was added to the upper layer. Then, 600 µL complete medium containing 10% FBS was added into lower chambers. These chambers were incubated in the humidified incubator for 24 h. The compartment was removed, and we gently wiped off the upper cells with a cotton swab. The migrative cells in the lower layer of the chamber were fixed with 4% paraformaldehyde for 10 min. After washing with PBS twice, the cells were stained with 0.05% crystal violet for 15 min. Photographs were taken for counting under a microscope.

### 4.8. RNA Extraction and Quantitation

Total RNA was extracted from cells using Trizol^®^ reagent (Invitrogen, Carlsbad, CA, USA) according to the manufacturer’s instructions. For cDNA synthesis, 1 μg or 4 μg of total RNA from each sample was reverse-transcribed using random primers and AccessQuick™ RT-PCR System (Promega, Madison, WI, USA). Quantitative real-time PCR was performed using the 2X SYBR Green qPCR Master Mix (BIMAKE, B21203, Houston, TX, USA) in the LightCycler480 PCR system (Roche, Basel, Switzerland). The primer sequences used were as listed (Table 1). Relative mRNA levels were calculated according to the ΔΔCT method based on CT values. The expression of the Actin Beta gene was used for normalization.

### 4.9. Flow Cytometry

Flow cytometric analysis of cell apoptosis was detected by using the Annexin V-FITC/Propidium Iodide (PI) Apoptosis Detection Kit (LiankeBio, Hangzhou, China). CRC cells were plated in 6-well plates in complete medium. DIM and 5-FU were dissolved into fresh medium to replace the old medium. Cells were treated by the drugs for 48 h, and then both supernatant and adherent cells were collected by trypsinization. The cell suspension used for analysis was prepared with 500 μL cold PBS containing around 1 × 10^6^ cells. Annexin V-FITC (10 μL) and PI (5 μL) were added, respectively, and stained at room temperature in the dark for 5 min. The pretreated cells were analyzed by flow cytometry (CytoFLEX, Beckman Coulter, Inc., Miami, FL, USA).

For cell cycle distribution analysis, a propidium iodide (PI) cell cycle staining kit (LiankeBio, Hangzhou, China) was used according to the manufacturer’s instructions. After being treated with various concentrations of DIM for 48 h, cells were washed with cold PBS three times and then trypsinized to investigate the distribution of the cell cycle phases by flow cytometry (CytoFLEX, Beckman Coulter, Inc., Miami, FL, USA).

ROS level was detected using reactive oxygen species assay kit (S0033S, Beyotime, Beijing, China). Generally, DLD-1 cell and HCT116 cell were treated with DIM for 48 h. Then, DCFH-DA was diluted with serum-free medium at 1:5000 to achieve a final concentration of 2 μM. The original medium was replaced with diluted DCFH-DA at a volume of 2 mL/well, and incubated at 37 °C for 20 min. The cells were washed three times with serum-free cell medium to fully remove the nonspecific binding DCFH-DA. Cells were analyzed using flow cytometry (CytoFLEX, Beckman Coulter, Inc., Miami, FL, USA). Data analysis with FLOWJO V10.6.2 software (Tree Star, San Francisco, CA, USA) was used to analyze the data and create the histogram.

### 4.10. TdT-Mediated dUTP Nick-End Labeling (TUNEL) Assay

Paraffined-embedded xenograft sections were subjected to TUNEL staining according to the manufacturer’s instructions. Briefly, paraffined-embedded xenograft sections were deparaffinized in xylene, rehydrated in graded ethanol, and washed with water before being treated with 20μg/mL Proteinase K (B600452, Sangon, Shanghai, China) for 15 min at 37 °C. Subsequently, they were washed with PBS 3 times and then stained with the TUNEL kit (C1086, Beyotime, Beijing, China) for 60 min at 37 °C in dark. After being stained with TUNEL solution, xenograft sections were counterstained with 4, 6-diamidino-2-phenylindole (DAPI, Invitrogen, Carlsbad, CA, USA) for 5 min. The apoptotic cells were detected, and the results were observed using a fluorescence microscope (Leica DMi8, Wetzlar, Germany).

### 4.11. RNA Sequencing

RNA-seq was carried out on the BGISEQ-500 platform using the single-end 50 bp protocol. DLD-1 cells were grown with DMSO or 40 μM DIM in RPMI medium 1640 for 48 h. Total RNA was harvested from DLD-1 cells after treatment. The mRNA with polyA tail was enriched by magnetic beads with OligodT. The RNA obtained was segmented by interrupting buffer, the random N6 primers were used for reverse transcription, and then the cDNA two-strand was synthesized to form double-stranded DNA. The end of the synthesized double-stranded DNA is blunted, and the 5’ end is phosphorylated, while the 3’ end forms a sticky end with an “A” protruding, and then a bubbling linker with a protruding “T” at the 3’ end is connected. The constructed cDNA library was inspected and sequenced after being qualified. The ligated products are amplified by PCR with specific primers. The PCR product was heat-denatured into single-stranded DNA, and then a single-stranded circular DNA library was obtained by circularizing the single-stranded DNA with a bridge primer. The constructed library is qualified by a standard cDNA library and sequenced after it is qualified. Subsequently, sequenced data filtering with quality score was performed on sequenced raw reads, and the process was performed as described [38]. GSEA software (Broad Institute, Cambridge, MA, USA) was used to operate gene set enrichment analysis to search for the biological change between groups.

### 4.12. Metabolite Profiling Isotope Analysis

CRC cells were prepared for metabolic analysis. HCT116 cells were seeded in 6-well plates. Cells were pretreated with DIM (40 μM) for 24 h in regular medium. Glucose-free RPMI-1640 medium or medium containing 11mM ^13^C-glucose (Cambridge Isotope Laboratories, Inc. Andover, MA, USA) were supplemented with 10% FBS and DIM to replace the regular medium for 24 h. Cells were harvested to extract the metabolites through 80% pre-cooled methanol after a total of 48 h of DIM treatment. Extracts were centrifuged at 15,000 rpm for 15 min at 4 °C. The supernatant containing metabolites was further dried under vacuum, and then the dried pellet was sent for mass spectrometry analysis. The Dionex UltiMate 3000 LC System (Thermo Scientific, Waltham, MA, USA) in equipped with a Q Exactive Orbitrap mass spectrometer (Thermo Scientific, Waltham, MA, USA), and the negative mode default parameters described previously were used for mass spectrometry analysis [43]. Natural isotopes have been corrected so that it is calculated as the total carbon contribution. The intensity of metabolite was further processed for plotting, using the protein content for normalization and relative to the internal reference.

### 4.13. In Vivo Studies Using Xenograft CRC Model and Treatment

Male BALB/c nude mice (5-to-6-week-old, 16–20 g) were purchased from GemPharmatech (Nanjing, Jiangsu, China). Flanks of the mice were subcutaneously injected with HCT116 cells (1 × 10^6^ cells/mouse) or DLD-1 cells (1 × 10^6^ cells/mouse). After several days, palpable tumors had developed, and mice were randomly divided into indicated groups. HCT116 xenograft mice were divided into two groups: the control group (*n* = 7) and DIM-treated group (*n* = 7). DLD-1 xenograft mice were divided into four groups: the control group (*n* = 6), DIM group (*n* = 6), 5-FU group (*n* = 6), and DIM + 5-FU group (*n* = 6). Mice were treated with DIM (40 mg/kg, 10% DMSO in corn oil) twice every 3 days by intraperitoneal (IP) injection, 5-FU (40 mg/kg, PBS) every 3 days by IP injection, or both DIM and 5-FU. The tumor length and width were measured every 3 days, and the mice were sacrificed 3 weeks after treatment. The volume was calculated according to the formula (length × width^2^)/2. All animal experiments were approved by the Animal Ethical and Welfare Committee of The Sixth Affiliated Hospital of Sun Yat-Sen University.

### 4.14. Statistical Analysis

Statistical analysis was carried out using the software GraphPad Prism 8.0 (GraphPad Software Inc., La Jolla, CA, USA). Heatmap was created by Sangerbox platform (http://vip.sangerbox.com/home.html, accessed on 27 April 2022), and other graphs were created by GraphPad Prism. Mean ± SEM was used to plot the data. Student’s *t*-test, one-way ANOVA test, and two-way ANOVA test were used to analyze quantitative data between groups. A value of *p* < 0.05 was considered statistically significant.

## 5. Conclusions

DIM is a potential anticancer compound, and our present study shows that DIM could enhance 5-FU chemotherapeutic efficacy in CRCs. Using a combination of RNA sequencing and metabolic profiling, we found that the underlying mechanism of DIM is the block in DHODH inducing the disruption of pyrimidine synthesis. Subsequently, we verified the synergistic effect of DIM with 5-FU in vivo and in vitro and found that DIM could enhance the antitumor efficacy of 5-FU. These results reveal the benefits of DIM in combination with 5-FU treatment, which could potentially allow us to introduce a novel strategy for the CRC patients who received 5-FU-based chemotherapy.

## Figures and Tables

**Table 1:** Primers for qPCR.

Gene Name	Forward Primer	Reverse Primer
CAD	CCATGCACTAGACAGCCAAGA	CGGCTCAGTGTGGATACGAC
DHODH	CCACGGGAGATGAGCGTTTC	CAGGGAGGTGAAGCGAACA
UMPS	TCTCGACCGCGTCTTCTGA	ACACACGGTGTCAAAACTGAT
NME1	AAGGAGATCGGCTTGTGGTTT	CTGAGCACAGCTCGTGTAATC
CTPS	CCTGGGTAACTATGAGCGGTT	ACAACTTGGACAGTTTTCCCC
RNR	ACTTCGGCTTTAAGACGCTAGA	GCATGAGTAAACCACCTCTCAGA
UPP1	GGTGCTCCAACGTCACTATCA	TTGAAGCAGGTATCCACTGCC
Actin Beta	CATGTACGTTGCTATCCAGGC	CTCCTTAATGTCACGCACGAT

## Data Availability

The data presented in this study are available in article.

## Abbreviations

Colorectal cancer	CRC
3,3′-diindolylmethane	DIM
5-fluorouracil	5-FU
Gene set enrichment analysis	GSEA
Dihydroorotate	DHO
Dihydroorotate dehydrogenase	DHODH
Cytidine triphosphate	CTP
Uridine triphosphate	UTP
Carbamoyl phosphate	Car-Asp
Fetal bovine serum	FBS
